# Strategies for the quality assessment of the health care service providers in the treatment of Gastric Cancer in Colombia

**DOI:** 10.1186/s12913-017-2440-8

**Published:** 2017-09-15

**Authors:** María del Pilar Villamil, David Barrera, Nubia Velasco, Oscar Bernal, Esteban Fajardo, Carlos Urango, Sebastian Buitrago

**Affiliations:** 1 Department of Systems and Computing Engineering, University of Los Andes, Cr 1E No. 19A-10, Bogotá, Colombia; 2 Department of Industrial Engineering, Pontificia Universidad Javeriana, Cr 7 No. 40 - 62, Bogotá, Colombia; 3 School of Management, University of Los Andes, Calle 21 No. 1-20, Bogotá, Colombia; 4 Escuela de Gobierno, University of Los Andes, Cr 1E No. 19A-10, Bogotá, Colombia; 5 Department of Industrial Engineering, University of Los Andes, Cr 1E No. 19A-10, Bogotá, Colombia

**Keywords:** Quality assessment, DEA, Process mining, Data mining, Clustering

## Abstract

**Background:**

While, at its inception in 1993, the health care system in Colombia was publicized as a paradigm to be copied across the developing world, numerous problems in its implementation have led to, what is now, an inefficient and crisis-ridden health system. Furthermore, as a result of inappropriate tools to measure the quality of the health service providers, several corruption scandals have arisen in the country. This study attempts to tackle this situation by proposing a strategy for the quality assessment of the health service providers (*Entidades Promotoras de Salud*, EPS) in the Colombian health system. In particular, as a case study, the quality of the treatment of stomach cancer is analyzed.

**Methods:**

The study uses two complementary techniques to address the problem. These techniques are applied based on data of the treatment of gastric cancer collected on a nation-wide scale by the Colombian Ministry of Health and Welfare. First, Data Envelopment Analysis (DEA) and the Malmquist Index (MI) are used to establish the most efficient EPS’s within the system, according to indicators such as opportunity indicators. Second, sequential clustering algorithm, related to process mining a field of data mining, is used to determine the medical history of all patients and to construct typical care pathways of the patients belonging to efficient and inefficient EPS’s. Lastly, efforts are made to identify traits and differences between efficient and inefficient EPS’s.

**Results:**

Efficient and inefficient EPS were identified for the years 2010 and 2011. Additionally, a Malmquist Index was used to calculate the relative changes in the efficiency of the health providers. Using these efficiency rates, the typical treatment path of patients with gastric cancer was found for two EPSs: one efficient and another inefficient. Finally, the typical traits of the care pathways were established.

**Conclusions:**

Combining DEA and process mining proved to be a powerful approach understanding the problem and gaining valuable insight into the inner workings of the Colombian Health System, especially in terms of the treatment process performed by health care providers in critical illnesses such as cancer. However, no sufficiently compelling results were found to establish the contribution of such a combination to evaluate the quality in the delivery of health services.

## Background

The Colombian Health System (CHS) is regulated by Law 100 of 1993. The CHS has evidence several problems in its implementation [1] and there is consensus across the country about the of an structural reform. Under these difficult circumstances, it is important to develop strategies and establish indicators that allow to estimate the quality of the health services in order to improve the system, which is of paramount importance for its population’s well-being and quality of life.

The heath care system is comprised of three independent actors. First, the institutions providing health services (IPS - *Instituciones Prestadoras de Servicios de Salud*) as hospitals, laboratories, health centers, among others; they are directly responsible for the treatment of patients and for providing all the necessary resources for the restoration of health and disease prevention. Second, the insurance companies known as Health Promotion Entities (EPS - *Entidades Promotoras de Salud*), they serve as intermediaries and administrators of the state resources. And third, the government, through the Colombian Ministry of Health and Welfare (MHW) and the Committee on Health Regulation (CHR), responsible for the regulation, control and management of the whole system.

As part of the compensation scheme the CHS, each IPS send to the EPS the minimum patient-data that the IPS are required to report in order to address, regulate and control the CHS. This data knows as Health Services Delivery Records RIPS (*Registros Individuales de la Prestación de Servicios de Salud*), includes, according to the law: user identification, type of consultation, type of procedures, emergency service data and medication and hospitalization data. The RIPS are issued at national-level and are consolidated and maintained by the MHW in a data warehouse.

The existence of RIPS enables the opportunity to measure the quality of the CHS, giving the possibility to identify strengths, weakness in the health system, and opportunities to improve the population’s well-being. These issues, in conjunction with the necessity of measure the system quality and the need to develop unbiased measures of efficiency in the IPS to ensure an efficient system [2], motivate this work.

This paper looks for evaluate the quality of the treatment for stomach cancer in Colombia, given that this type of cancer was one of the top thirteen causes of cancer-related death in the country during the year 2015 [3], according to the Colombian epidemiological monitoring group on cancer. The proposal presents a strategy to evaluate the quality of gastric cancer treatment procedures in Colombia using DEA and process mining techniques. Firstly, quality indicators for each EPS are estimated. Secondly, a DEA model and the Malmquist Index are used to calculate the efficiency and the change in efficiency between 2010 and 2011, for a given institution. Finally, using sequential clustering, a typical algorithm in process mining, care paths for all patients are identified in order to assess the quality of a particular treatment. Furthermore, the proposed strategy can be used to identify the care paths for efficient and inefficient institutions, in order to compare them.

The paper is structured as follows: “Methods” section gives an overview of the methods used in the study and explores quality assessment in the context of gastric cancer. “Results and discussion” section presents the applicability of process mining and DEA to the treatment of gastric cancer in Colombia and presents our results. Finally, future work directions and conclusions are presented in “Conclusions” section.

## Methods

The Colombian mandatory system of quality assurance (SOGCS, *Sistema Obligatorio de Garantía de Calidad en Salud*) includes a series of methodological elements to assess the quality of the health services. The current legislation states that the evaluation of the quality of services must be made by a comparison of observed quality and expected quality [4]. Such quality is validated based on pre-established rules, which are known to all participants in the health care system. These rules can come in different formats: manuals, practice guides, technical standards, and indicators, among others.

In this study, we chose to focus on the concept of clinical guidelines, which is defined as "a set of systematically developed recommendations to help practitioners and patients to make health care decisions and to select the most appropriate diagnostic and treatment options when addressing a health problem or specific condition" [5].

In order to evaluate the quality of gastric cancer treatments, we propose a three step strategy. First step prepares the information from RIPS to obtain those that are related to gastric cancer and with reliable information. Second step consists on applying DEA and the Malmquist Index techniques over the available information from different EPS. These techniques are applied by using a set of previously defined quality indicators related to the diseases treatment as base.

Finally, the third step selects a set of EPS with distinct efficiency characteristics and analyzed their treatment processes using the sequential clustering algorithm to construct typical care pathways. These efforts were aimed at identifying tendencies and patterns regarding the treatments applied to patients and relate them to the quantitative efficiency measures previously gathered for efficient and inefficient EPSs.

### Data preparation

Data preparation consists of three basic steps: 1) Selection of patients diagnosed with gastric cancer; 2) Analysis of the quality of the available data; and 3) Grouping of the procedures performed on the patients starting from the time of the diagnosis.

*Patient Identification.* Table 1 shows stomach cancer-related diseases codes used by ICD-10 standard (International Statistical Classification of Diseases and Related Health Problems 10th Revision). This standard is the one used on the RIPS an allows to identify all the patients suffering from stomach cancer in Colombia.

**Table 1:** ICD-10 codes and number of patients

Code	Description	# Patients
D131	Benign neoplasm of stomach	176
Z120	Special screening examination for neoplasm of stomach	129
C160	Malignant neoplasm of cardia	80
C161	Malignant neoplasm of fundus of stomach	559
C162	Malignant neoplasm of body of stomach	363
C163	Malignant neoplasm of pyloric antrum	108
C164	Malignant neoplasm of pylorus	18
C165	Malignant neoplasm of lesser curvature of stomach	101
C166	Malignant neoplasm of greater curvature of stomach	75
C168	Malignant neoplasm of overlapping lesion of stomach	35
C169	Malignant neoplasm of stomach, unspecified	1618
D000	Carcinoma in situ of lip, oral cavity and pharynx	54
D001	Carcinoma in situ of esophagus	104
D002	Carcinoma in situ of stomach	357

*Analysis of the quality of the data*. The patient data and their related procedures are analyzed in order to validate data quality. The two most common quality-related problems for the patients are data replication and inconsistencies between the procedures performed and the diagnosis; 9.9% of patients diagnosed with cancer are reported more than once between 2010 and 2011. Additionally, 1.2% of these patients did not have surgical procedures between 2010 and 2011, and only 39% of procedures had a patient associated to them in the analyzed data. All records with one of the aforementioned problems were excluded from the analysis.

*Grouping of procedures*. In order to reduce the number of different procedures to be analyzed and as suggested by an oncologist, a filter was applied based on the CUPS classification (*Clasificación Única de Procedimientos en Salud*). The CUPS, according to Resolution 1896 of 2001 of Colombia, are a logical, hierarchical and detailed classification of health procedures and interventions performed in Colombia, identified by a code and described by a unique nomenclature. The analyzed procedures in this work are related to the first three characters as follows:

*Selected first-level group filter*

- Digestive System
- Imaging
- Consultation, Monitoring and Diagnostic Procedures
- Clinical Laboratory
- Transfusiology and Blood Bank
- Nuclear Medicine and Radiation Therapy
- Other nonsurgical procedures
- Miscellaneous Procedures

*Selected second-level subgroup filters*

- Transfusiology and Blood Bank
- Radiological Imaging
- Clinical Laboratory
- Nuclear Medicine and Radiation Therapy +
- Stomach Related Procedures
- Intestine Related Procedures
- Procedures in the Abdominal Wall
- Esophagus procedures
- Prophylactic, therapeutic and other miscellaneous procedures

As in the precedent step, data quality-related problems were detected: *(1)*difficulty to validate the relationship between the procedure and the analyzed disease, *(2)*Data replication, and *(3)* reliability in the content of the attributes.

13.31% of records presented problems insofar as identifying a relationship between the procedure used and the disease analyzed. Approximately, 65% of procedures can be used for different health problems, affecting the precision in the analysis. Additionally, there are procedures related to the same patient on the same date, or a near date that are not possible. For example, complete stomach surgery followed by partial stomach surgery. Finally, the most important cases regarding reliability in the content of the attributes are related to time. In fact, the majority of records have 00:00:00.000 as their time value. All records with the aforementioned problems were identified and excluded.

### Data envelopment analysis

Data Envelopment Analysis (DEA) is a non-parametric efficiency approach. It was developed by [6] and later elaborated by [7] (BCC model). DEA computes the relative efficiency of decision-making units (DMUs) with many inputs and outputs [8]. In the same way, DEA allows the quality assessment to include not only a set of performance indicators (outputs) but also the amount of consumed resources (inputs). As a result, DEA it is now considered mainstream to appraise the efficiency of health institutions [2]. Furthermore, Emrouznejad et. al in [9] concluded that DEA applications will continue to be a primary arena of research in the future.

In operations management, DEA is used for benchmarking where a set of measures (indicators) is selected to estimate the performance of production and/or service operations, comparing multiple DMUs with a structure of multiple inputs and outputs. As a result, a set of DMUs that belong to a “best-practice frontier” [10], are identified. This frontier, allows us to calculate an efficient solution for every level of input or output. Any DMU not on the frontier is considered inefficient. A numerical coefficient is given to each firm, defining its relative efficiency. Where there is no actual corresponding firm, virtual efficient producers are identified to make comparisons [11].

Classical DEA models rely on the assumption that inputs have to be minimized while outputs have to be maximized [12]. However in health care, one or more outputs -called undesirable outputs- have to be minimized [13]. Even more, according to [14] considering such variables, in efficiency analysis, have paved the way for more thorough assessments. Nevertheless, modeling undesirable outputs has been object of considerable discussion in the efficiency literature, because of the lack of consensus about the most appropriated approach [3]. Even when variable transformations can be addressed to avoid this problem, [15] conclude that such transformations could generate loss of linearity. Authors also compare methods to deal with this situation.

Liu et al. [16] presented a survey of DEA applications from 1978 and 2010. According to the authors, health care is the second largest application area. Authors also state that most of the reviewed papers studied hospital performance. More recent papers studied the integration of DEA and complementary techniques to measure health care efficiency. As an example, Al-Refaie et al. [17] applied simulation and DEA to improve the emergency department of a Jordanian hospital. In this research DEA was used to identify the best possible scenario regarding nurses’ workload. They concluded that using DEA to develop quality frontiers in health services is a new promising direction.

In recent years, several studies to evaluate health policies and health services has been developed. In Uganda, DEA was used to evaluate the efficiency of referral hospitals [18], as the same in Angola [19], Zambia [20], South Africa [21], among others. In Asia, recently [22] propose to evaluate the performance of maternal and child services in China hospitals using DEA, comparing poverty and non-poverty country hospitals. In Latin-America there are few studies that use DEA to evaluates performance in health institutions [23] propose DEA as new strategy to evaluate efficiencies in Chilean hospitals. In the best of our Knowledge there are not studies that allow us to evaluate the quality of a procedure in Colombia.

To evaluate the quality of gastric cancer treatments in different Colombian EPSs, a DEA model with non desirable output variables keeping the linearity between them are considered. After analyzing different models, we use the model proposed by [15], an output-oriented model with variable return to scale. The mathematical model is the following:

1 $$ \max \rho_{0}  $$

Subject to:

2 $$ \sum\limits_{j=1}^{n}{\eta_{j} x_{ij} + s_{i}^{-}}=x_{i0} \ \ \ i=1 \ldots m  $$

3 $$ \sum\limits_{j=1}^{n}{\eta_{j} y_{rj} - s_{r}^{+}}=\rho_{0} y_{r0} \ \ \ r=1 \ldots s   $$

4 $$ \sum\limits_{j=1}^{n}{\eta_{j} b_{tj} - s_{t}^{+}}=2b_{t0}-\rho_{0}b_{t0} \ \ \ t=1 \ldots T   $$

Let *x*, *y* and *b* be sets that represent input desirable variables, output desirable variables and output non desirable variables. Equation 1 is the objective function that looks to maximize efficiency considering the constrains over each set of variables, represented by Eqs. 2 to 4. This last set of equations, are used to assign weights to output and input variables and to estimate the distance of each EPS from the efficient frontier.

[24] Productivity Index was developed to measure changes in technological productivity over a period of time. This index was initially proposed by [24] and has been used in several studies with multiple variants such as [25, 26]. In order to measure the changes of EPSs’ efficiency over the observed period, we used the index used by [27], defined as follows:

5 $$ MI=\left[\frac{\delta^{1}\left.\left(\left(x_{0},y_{0}\right)^{2} \right)\right)}{(\delta^{1}((x_{0},y_{0})^{1})} \times \frac{\delta^{2}\left.\left(\left(x_{0},y_{0} \right)^{2} \right)\right)}{(\delta^{2}((x_{0},y_{0})^{1})}\right]\frac{1}{2}  $$

Let *δ* ^(^ *t*_2_)((*x*_0_,*y*_0_)^(^ *t*_1_)) be the efficiency of one DMU (*x*_0_,*y*_0_)^(^ *t*_1_) measured with respect to the technological frontier *t*_2_, and obtained from the results of the DEA model.

From the RIPS and based on health professionals’ opinions, the following efficiency indicators were calculated for each EPS and used for the DEA analysis:

- Input variables:
  - Number of patients: The total number of newly diagnosed patients with gastric cancer and, at least, one associated treatment procedure as classified by the EPS, for the period of study. A treatment procedure may be a surgical procedure associated with cancer, chemotherapy or radiotherapy. This indicator is taken as a desirable input variable for the model
- Output variables
  - Treatment opportunity: The average number of days per patient between the time of diagnosis and the first procedure for the treatment of the disease, as classified by the EPS. This indicator is taken as a desirable input variable for the model.
  - Number of readmissions: The average number of readmissions per patient, as classified by the EPS, for patients who had at least one emergency readmission after undergoing surgery in the study period. This indicator is taken as an undesirable output variable.
  - Number of previous studies: The average number of diagnostic studies prior to the first diagnosis of cancer per patient as classified by the EPS. This indicator is taken as a desirable output variable.
  - Number of histological studies: The average number of histological studies per patient, as classified by the EPS. This indicator is taken as a desirable output variable.

Once the input and output variables were defined, the most (and least) efficient EPSs in the Colombian Health System were identified. Subsequently, the efficient frontier was computed using the calculated efficiencies of each institution. Finally, the Malmquist indicator for each institution was calculated to evaluate the change in efficiency in the study period.

### Process mining

Process mining is a field of data mining that allows the discovery of business processes in a given domain using different types of algorithms. Examples of process mining algorithms include genetic algorithms, heuristics mining and sequential clustering, among others [28]. This work uses a ProM plugin that provide the Sequence Clustering Algorithm, a combination between sequence analysis (first-order Markov chain) and clustering. In particular, we decided to use sequential clustering, given its popularity in the process mining community, and information provided to interpret the results. In fact, sequential clustering algorithms generate a series of discrete states that are very similar internally [29]. These series, known as clusters are represented by a Markov chain consisting in states and transition probabilities between them. These probabilities depend only on the current state.

The use of process mining in this work allowed us to identify typical treatments used in patients by distinct EPSs to analyze various treatment patterns and, in turn, improve the quality of the health services. To do this, we had to carefully select the EPSs to be analyzed and the model to be used to do this, as well as prepare the data to execute the model, and evaluate the results.

This project uses two different EPSs: the most and least efficient according to DEA results. This selection allows a comparison to be made between these EPS to understand patterns affecting treatment quality. On the other hand, the design of the model consists in creating the sequential clustering model, and preparing the data used in the analysis related to procedures applied in treatments. It is important to note that a detailed granularity level of information used can increase the complexity of the results because of the number of procedures and relations between them. Finally, the evaluation, presented in the following “Evaluation” section includes the criteria of an oncologist to validate the quality of the identified treatments.

#### Data models

The data model used in the study includes two entities that consolidate information from patients with gastric cancer and procedures used in the treatment of these patients. According to patients, the entity has a unique patient identifier, information about institutions related to the health service (EPS and IPS unique codes), date of the first diagnosis, main diagnosis of the consultation, and other three possible diagnoses related to the consultation. On the other hand, the entity where the procedure takes place holds information about the date of procedure, institutions related to the health service (EPS and IPS unique codes), patient identifier, procedure code (CUPS identifier), and the main diagnosis of the procedure.

The entity where the procedure takes place does not hold information on time, given the previously discussed data quality problem in this field. As a consequence, the identified paths using sequential clustering represent patients immediately operated on after the diagnosis, although, in reality, that is not the case.

#### Data aggregation

An aggregation strategy based on the CUPS was used in order to reduce the complexity of the models arising from the vast number of different treatments that a cancer patient can be subjected to and to facilitate analysis by the experts, according to procedure characteristics such as: type of procedure (diagnostic, radiotherapy, surgery) and affected organ (intestine, stomach). Table 2 shows the aggregation scheme proposed to characterize non-surgical and surgical procedures, presented in the CUPS section column. This schema has two levels to define the procedures, **Level 1** and **Level 2** columns in the Table. According to non-surgical procedures, Level 1 represents diagnosis, radiotherapy and chemotherapy procedures. Level 1 for surgical procedures represents diagnosis, pre and post operative and surgery procedures. Level 2 has more specific information about a Level 1 procedure. For example, non-surgical diagnosis procedures are classified in terms of radiography, tomography, clinical examination, derived examination and scintigraphy. Finally, the percentage in brackets in the Table represents the percentage of procedures in each category used in this work. In this way, the information used contains 92.2% of non-surgical procedures.

**Table 2:** Two-level aggregation scheme

CUPS section	Level I	Level 2
		Radiography (7,72%)
		Tomography (5,06%)
	Diagnosis (86,3%)	Clinical Examination (45,7%)
Non-surgical		Derived Examination (27,2%)
Procedures (92,2%)		Scintigraphy (0,55%)
	Radiotherapy (0,5%)	Teletherapy and Therapy with radioisotope (0,5%)
	Chemotherapy (5,3%)	Chemotherapy (5,3%)
	Diagnosis (5,2%)	Biopsy (0,08%)
		Cavity Exploration (5,1%)
Surgical	Pre-Post Operative (1,5%)	Pre-Postoperative (1,5%)
Procedures (8,2%)		Abdominal (0,08%)
		Esophagus (0,17%)
	Surgery (1,6%)	Stomach (1,18%)
		Intestine (0,13%)

#### Evaluation

The evaluation phase is carried out by a gastric cancer oncologist, according to whom, the quality of a given medical treatment depends on some of the features included in the patient’s care pathway. Thus, the treatments administered to a patient with gastric cancer should include the following features:

- Will follow the treatment established in the clinical guide for the disease.
- Will establish diagnostic procedures before and after a surgical procedure.
- Will include procedures such as chemotherapy or radiation treatments. Furthermore, these procedures should be applied sequentially.

## Results and discussion

**Fig. 1:**
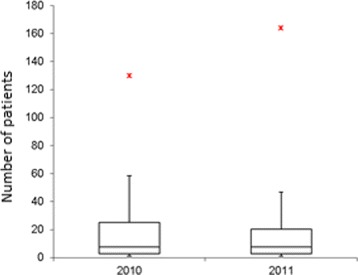
Number of patients. Years 2011 and 2012

**Fig. 2:**
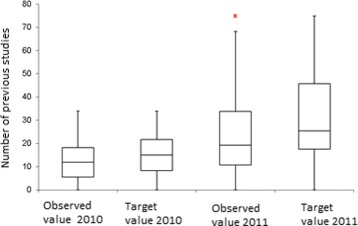
Comparison between the number of previous studies and the target

**Fig. 3:**
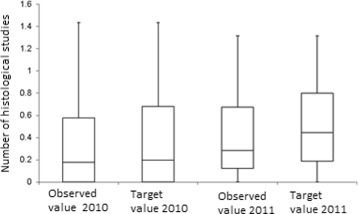
Comparison between the number of histological studies and the target

**Fig. 4:**
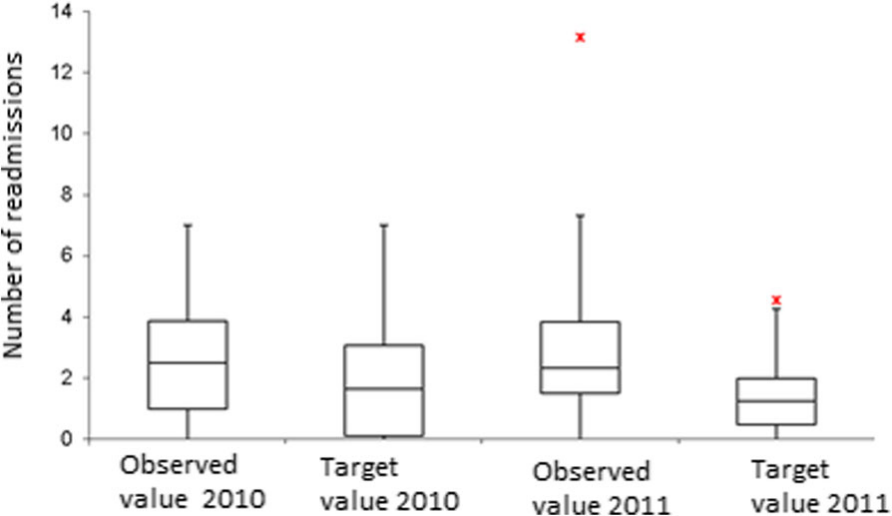
Comparison between the number of readmissions studies and the target

**Fig. 5:**
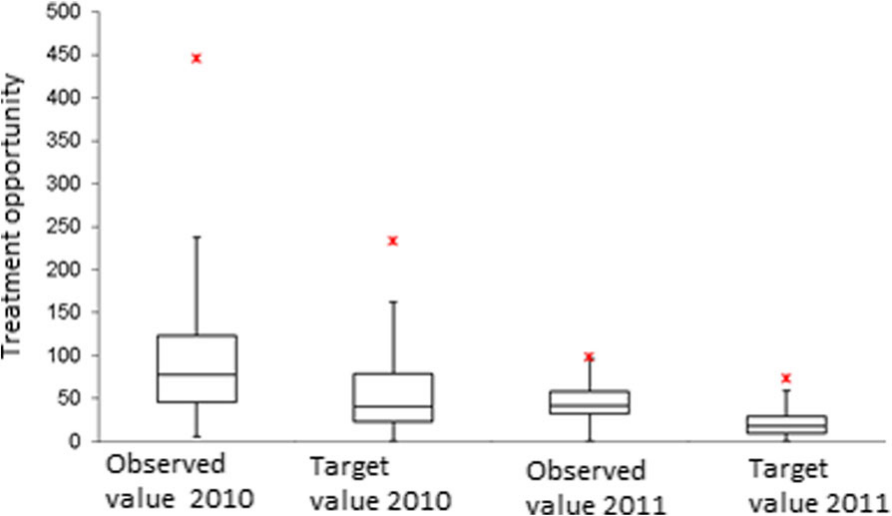
Comparison between the treatment opportunity and the target

The methodology described above, is tested using information from the reported RIPS in 2010 and 2011. The efficiency indicators are estimated for each EPS, for each year. Figures 1, 2, 3, 4 and 5 summarize those results. Each figure indicates the target value (for 2010 and 2011), the highest value (asterisk) and four quartiles.These figures show the high variability among EPS for each indicator.

From the same figures, we can conclude that the variability of indicators decreased from 2010 to 2011, except in the case of “number of previous studies” (Fig. 2). Figure 5 shows how the treatment opportunity decreased from 2010 to 2011, reflecting the effort made for each EPS to diminish the time elapsed between the diagnoses and the beginning of treatment.

Using a mean differences hypothesis tests, with unknown variances, it is possible to say that there is no statistical evidence to affirm that there are differences in histological studies and in the number of readmissions to EPS. On the other hand, it is possible to reject the null hypothesis of average difference for treatment opportunity and previous studies indicators, which present improvements in the observation period.

### DEA

The proposed DEA model is implemented on Xpress-MP. The results of the efficiency and target values for each EPS in 2010 and 2011 are shown in Tables 3 and 4 respectively. For 2010, 27% of the EPS are considered efficient and 24% extremely inefficient (i.e., efficiencies below 60%). To become efficient, each EPS belonging to quantile 4 must improve its indicators by up to 80%. For example, they have to reduce the treatment opportunity from 185 days to 11 days, and readmissions from an average rate of 1.8 to 0.13. Finally, the results show that some EPS on the best-practice frontier have worse indicators than the ones that are fixed as target values for inefficient EPS. For instance, EPS 3 has 13.67 days of treatment opportunity; however, for 5 of the 7 inefficient EPS, target values are below that value. This can be explained by the variability among the indicators; while EPS 3 has a poor performance at treatment opportunity, it has notable indicators in readmissions and previous studies.

**Table 3:** Results 2010

ID	Efficiency	Patients	Previous studies		Histological studies		Re-admissions		Treatment opportunity	
		Observed	Observed	Target	Observed	Target	Observed	Target	Observed	Target
1	1	1	5	5	0	0	7	7	6	6
2	1	1	3	3	1	1	0	0	33	33
3	1	3	10,33	10,33	0	0	0	0	13,67	13,67
4	1	4	32,5	32,5	0,75	0,75	2,5	2,5	128,5	128,5
5	1	5	25	25	0	0	1,33	1,33	98,6	98,6
6	1	28	33,96	33,96	0,21	0,21	3,13	3,13	78,36	78,36
7	1	23	16,83	16,83	1,43	1,43	4	4	77,65	77,65
8	1	45	19,02	19,02	0,69	0,69	3,27	3,27	39,04	39,04
9	0,962	7	17,71	18,41	0,14	0,15	2,5	2,4	34,43	35,96
10	0,954	3	9	9,44	0,67	0,67	6	5,71	31,67	30,13
11	0,949	8	30,25	31,86	0	0	2,83	2,68	92	87,1
12	0,914	76	26,14	28,62	0,59	0,65	4,45	4,03	93,08	84,28
13	0,863	130	12,12	14,05	0,66	0,77	3,41	2,87	43,52	36,6
14	0,828	26	13,12	15,84	0,54	0,65	2,25	1,78	55,81	44,2
15	0,822	29	11,62	14,13	0,76	0,92	4,2	3,29	57,38	44,98
16	0,813	32	18,38	22,61	0,25	0,31	5,04	3,88	62,09	47,78
17	0,811	13	11,77	14,52	0,31	0,38	1	0,77	53,92	41,33
18	0,797	8	14,25	17,88	0,38	0,47	1,33	0,99	105,63	78,72
19	0,782	9	21	26,84	0,22	0,28	2,33	1,68	161,44	116,54
20	0,681	16	16,63	24,41	0,06	0,09	3,5	1,86	98,63	52,47
21	0,677	1	7	10,33	0	0	0	0	445	233,1
22	0,615	34	12,38	19,01	0,12	0,18	3,43	1,59	81,03	37,68
23	0,649	1	10	15,4	0	0	1	0,46	182	83,78
24	0,54	21	6,95	12,87	0,05	0,09	4,43	0,66	139,48	20,78
25	0,537	2	0	0	0,5	0,93	1	0,14	224	30,74
26	0,523	5	2,6	4,97	0	0	1	0,09	74,2	6,57
27	0,508	4	4	7,88	0	0	0	0	346,25	10,42
28	0,506	2	3,5	6,92	0	0	0	0	392,5	9,15
29	0,503	6	0,17	0,33	0	0	2	0,02	43,5	0,44
30	0,5	3	0	0	0	0	4,33	0	75,67	0

**Table 4:** Results 2010

ID	Efficiency	Patients	Previous studies		Histologial studies		Readmissions		Treatment opportunity	
		Observed	Observed	Target	Observed	Target	Observed	Target	Observed	Target
1	1	1	9	9	1	1	1	1	32	32
21	1	1	55	55	0	0	0	0	11	11
28	1	1	36	36	1	1	1	1	18	18
6	1	7	74,86	74,86	0,29	0,29	2	2	38	38
7	1	19	24,84	24,84	1,32	1,32	2,8	2,8	73,21	73,21
8	1	32	26,25	26,25	1,22	1,22	2,4	2,4	36,56	36,56
23	0,9	2	2	2,22	0	0	1,5	1,33	0,5	0,44
17	0,888	14	42,21	47,54	0,71	0,8	2,25	1,97	37,93	33,17
3	0,846	3	13,67	16,15	0,67	0,79	2	1,64	17,33	14,18
13	0,822	164	20,26	24,65	0,84	1,02	2,81	2,2	25,52	20
18	0,803	2	47	58,51	0,5	0,62	6	4,53	82	61,92
15	0,765	15	18,47	24,16	0,84	1,13	3	2,08	42,4	29,34
30	0,705	8	42,63	60,46	0,13	0,18	1,33	0,78	48	27,92
12	0,679	112	35,82	52,74	0,47	0,7	4,37	2,31	54,95	29
9	0,64	21	23,9	37,35	0,29	0,45	2,8	1,23	28,1	12,29
5	0,634	5	42,2	66,56	0	0	4,5	1,9	63,2	30,72
20	0,633	34	15,88	25,1	0,68	1,07	3,32	1,39	97,47	40,92
14	0,604	45	26,33	43,58	0,38	0,63	1,9	0,66	54,51	18,82
26	0,6	3	13,67	22,78	0	0	0	0	13,67	4,56
19	0,594	8	27,5	46,29	0,38	0,63	4	1,27	58,88	18,66
11	0,588	3	17,33	29,5	0,33	0,57	4	1,19	40,33	12,03
29	0,567	5	10	17,65	0,2	0,35	1,5	35	34	8
4	0,563	4	8,25	14,67	0,25	0,44	2	0,44	85,5	19
27	0,563	8	10	17,78	0,13	0,22	1	0,22	64	14,22
22	0,562	29	14,38	25,57	0,14	0,25	7,44	1,65	35	7,76
16	0,557	32	24,66	44,3	0,09	0,17	13,16	2,67	52,56	10,68
10	0,533	5	0	0	0,2	0,38	3	0,38	73	9,13
25	0,529	11	6,45	12,2	0,18	0,34	5	0,55	56,18	6,19
24	0,528	4	13,25	25,08	0	0	2	0,21	46,75	5,02
2	0,521	1	9	17,26	0	0	0	0	42	3,45

For 2011, the target values for all indicators were improved: the treatment opportunity indicator was reduced from 54 days in 2010 to 22 days in 2011; the previous studies indicator changed from 15 in 2010 to 32 for 2011. These results show the need to establish expected minimum (or maximum) values for each indicator. For example, even if the treatment opportunity target value was reduced to 45 days, reflecting an improvement for the patients’ health, this value is still higher than expected.

Considering the target values obtained for 2011, an overall improvement is observed: 20% of the EPS were efficient, and 37% of the EPS showed efficiencies below 60%. While in 2010, one "efficient EPS" had a treatment opportunity of 59 days and a 2.65 readmissions rate, for the same EPS, these values changed to 35 days and 1.53 readmissions. Finally, four of the efficient EPS in 2010, were also efficient during 2011.

On the other hand, the Malquimst index allows for identifying the effect of EPS policies over efficiency. For the EPS 21, for example, efficiency increased from 0.67 in 2010 to 1 in 2011 (see Table 5). Target values show that, EPS21 must increase previous studies from 7 (2010) to 10.3 (2011), and decrease treatment opportunity from 445 to 233 days. However, in 2011, the EPS21 registers 55 studies and 11 days treatment opportunity. Since, the improvement in efficiency of EPS 21 is not due to changes in the frontier (given by the target values) but to its own policies, the Malquimst index is equal to 3.96. It is important to note that for one EPS with only one diagnosed patient, making changes of this magnitude in the indicators requires less investment than the one required for one EPS with 164 patients.

**Table 5:** Malmquist index

ID	Malquimst index	Efficiency	
		2010	2011
21	3,96	0,677	1
28	3,13	0,506	1
23	2,82	0,649	0,9
30	2,17	0,5	0,705
6	2,13	1	1
26	1,74	0,523	0,6
17	1,69	0,811	0,888
18	1,48	0,797	0,803
8	1,47	1	1
3	1,46	1	0,846
13	1,42	0,863	0,822
1	1,38	1	1
29	1,31	0,503	0,567
9	1,25	0,962	0,64
5	1,24	1	0,634
27	1,23	0,508	0,563
24	1,22	0,54	0,528
15	1,21	0,822	0,765
12	1,21	0,914	0,679
22	1,21	0,615	0,562
19	1,19	0,782	0,594
20	1,19	0,681	0,633
14	1,13	0,828	0,604
16	1,12	0,813	0,557
7	1,04	1	1
11	1,03	0,949	0,588
25	0,89	0,537	0,529
4	0,67	1	0,563
10	0,66	0,954	0,533
2	0	1	0,521

### Process mining

This work used ProM 5.2 [30] as Process Mining tool, an open-source framework licensed under Common Public License (CPL), which allows the use of a variety of algorithms and friendly interface, in particular, sequential clustering.

The sequential clustering needs to configure a number of parameters such as: minimum and maximum percentage of events that each cluster can have (assigned values were respectively 0, 100), minimum and maximum number of different events that a sequence can have (we use respectively 1 and 303), minimum and maximum number of sequences that a cluster can have (we use 1 to 102 values), and number clusters that the model should contain, we decide to use 1. It should be noted that the purpose of this work was to try to identify a general pattern of treatment for all the patients in a specific EPS. To do this, it was decided to use a single cluster for EPS to represent the entire treatment process.

According to “Methods” section, two types of EPS were chosen to study and to illustrate the methodology, using the efficiency scores obtained from the DEA process.

1. **EPS 1:** This EPS is close to the efficient frontier and has a major positive change in the relative efficiency between the periods of study.
2. **EPS 2:** This EPS is well below to the efficient frontier and has a relatively low efficiency in the periods of study.

Table 6 shows the number of patients, procedures and efficiency scores of the two chosen EPSs.

**Table 6:** Number of patients, procedures and efficiency scores of the two chosen EPS

	Number of patients			Number of procedures			Efficiency	
	2010	2011	Total	2010	2011	Total	2010	2011
EPS 1	242	350	592	7425	9253	16678	0,863	0,822
EPS 2	102	84	186	1566	1185	2751	0,651	0,562

### EPS 1 (Close to the efficient frontier)

**Fig. 6:**
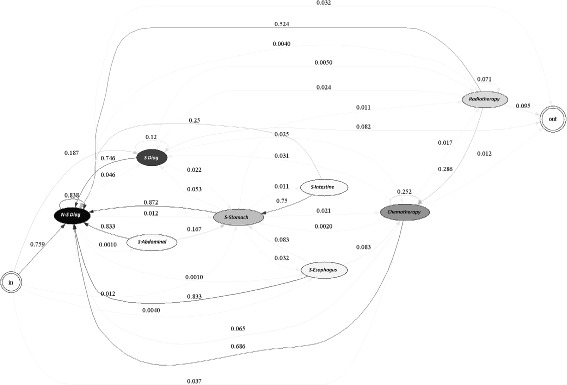
Typical Care Pathways for EPS 1, year 2010

**Fig. 7:**
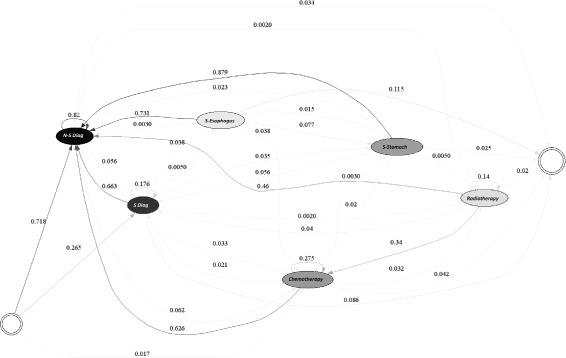
Typical Care Pathways for EPS 1, year 2011

Figures 6 and 7 demonstrate the typical treatment process for EPS 1 for each of the years studied. It is not possible to identify a general or distinctive path representing the treatment followed by patients in this EPS due to the complexity of the resulting model. This may be due to several reasons. First, a clinical guide for the treatment of gastric cancer may not be available or, this EPS may not follow it. In addition, it is also possible that the conditions during patient diagnosis are greatly varied, leading to a high number of possible treatments.

However, it is possible to discern paths in the treatment used by the EPS. For example, in the year 2010, the treatment usually started with either a non-surgical diagnostic procedure (75.9% of the time) or a surgical diagnosis procedure (18.7%). If a non-surgical diagnosis procedure was performed, usually an identical procedure was executed (82% of the time); then, the patient might have received chemotherapy treatment (6.5%) or a second operation (1.2%). Similarly, if a surgical diagnostic procedure was applied, typically a non-surgical diagnosis (74.6%) followed. Afterwards, the patient may have undergone chemotherapy treatment (2.5%) or a second operation (2.2%). Finally, when a patient underwent either surgery, chemotherapy or radiotherapy a diagnostic procedure normally followed.

Similarly, in the year 2011, the treatment usually started with either a non-surgical diagnostic procedure (71.8%) or a surgical diagnosis procedure (26.5%). If a non- surgical diagnosis procedure was performed, usually an identical procedure was executed (82%); then, the patient might have undergone chemotherapy treatment (6.3%) or entered into remission (3.4%). Likewise, if a surgical diagnostic procedure was applied, typically, a non-surgical diagnosis (66.3%) followed. Alternatively, the patient may have been subjected to chemotherapy treatment (3.3%) or a second operation (2.2%).

**Fig. 8:**
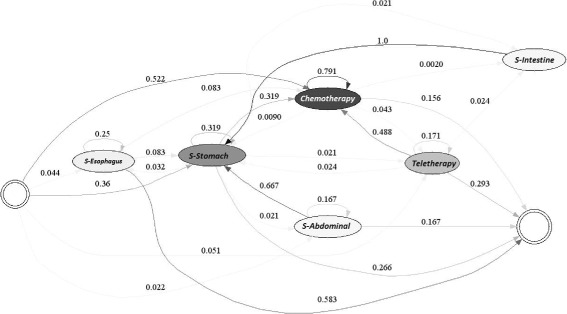
Chemotherapy / Radiotherapy Cycle for EPS 1, year 2010

**Fig. 9:**
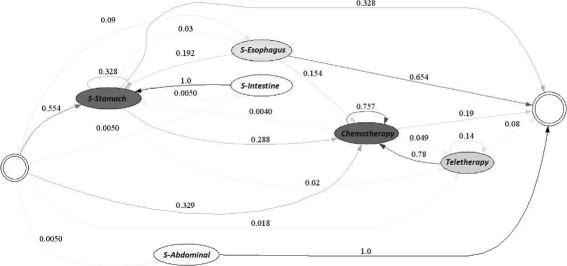
Chemotherapy/Radiotherapy Cycle for EPS 1, year 2011

Figures 8 and 9 exhibit the chemotherapy and/or radiotherapy cycle for each of the years studied. It should be noted that cycles of chemotherapy and radiotherapy were observed in the treatment of the patients, since 79.1% of those that received chemotherapy at least once, received chemotherapy again. The next most common procedure for the patients was radiotherapy, in 4.3% of the cases. Additionally, if a patient received radiotherapy, in 17.1% of the cases, she or he received another radiotherapy treatment and in 48.8% of the cases, the patient underwent chemotherapy.

EPS 1 diagnosed its patients in a timely, and effective manner, since its treatment traits are those identified above as those of a high-quality procedure for gastric cancer. As a consequence, the position of the EPS on the efficient frontier is not surprising, given that the treatment of its users follows one of the most appropriate for such patients.

### EPS 2 (Far from the efficient frontier)

**Fig. 10:**
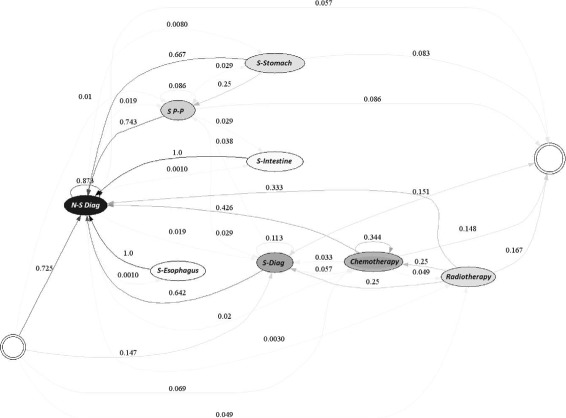
Typical Care Pathways for EPS 2, year 2010

**Fig. 11:**
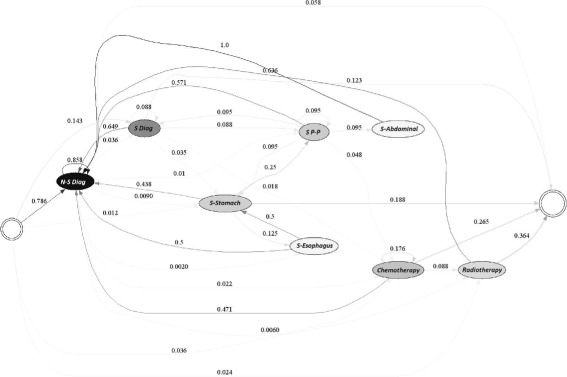
Typical Care Pathways for EPS 2, year 2011

Figures 10 and 11 show the typical treatment process for EPS 2 for each of the years studied. The resulting model is very complex as is the case for EPS 1. Hence, it is not possible to identify a distinct path representing the treatment of the patients, even though the number of patients was less than half of the patients for EPS 1.

However, it is possible to discern particularities in the treatment that the EPS performed. For example, in the year 2010, 75.9% of the time the treatment started with a non-surgical diagnosis, 14.7% with a surgical diagnosis and 12% with chemotherapy or radiotherapy. If a non-surgical diagnosis procedure was performed, usually an identical procedure was executed (87% of the times), and the patient probably ended her or his treatment (5.7%), 2% might have undergone chemotherapy or a second surgery (1.9%). Similarly, if a surgical diagnostic procedure was applied, typically a non-surgical diagnosis (64.2%) followed, after which the patient may have ended her or his treatment (15.1%), had a second surgical operation (11.3%) or received chemotherapy (5.7%). Finally, when a patient underwent either surgery, chemotherapy or radiotherapy a diagnostic procedure normally followed.

Similarly, during 2011, the treatment usually started with either a non-surgical diagnostic procedure (78.6%) or a surgical diagnosis procedure (14.3%). If a non-surgical diagnosis procedure was performed, usually an identical procedure was executed (85.8%), then, the patient might have received chemotherapy treatment (2.2%) or a surgical diagnosis. Similarly, if a surgical diagnostic procedure was applied, typically a non-surgical diagnosis (64.2%) followed. Alternatively, the patient may have received chemotherapy treatment (5.7%) or a second surgery (11.3%).

Consequently, as we can see from the analysis of EPS 1, it is clear that EPS 2 performs more surgeries than its counterpart. Similarly, the likelihood of a patient undergoing a second surgery soars in comparison with EPS 1. However, it should be pointed out that in most cases, surgical and non-surgical diagnostics were performed before procedures such as surgeries, chemotherapy or radiotherapy, showing that the treatment provided had at least a few desirable traits. Interestingly, unlike EPS 1, it was detected that 12% of the treatments began with a diagnosis that did not belong to the suggested clinical guidelines for the treatment of gastric cancer.

**Fig. 12:**
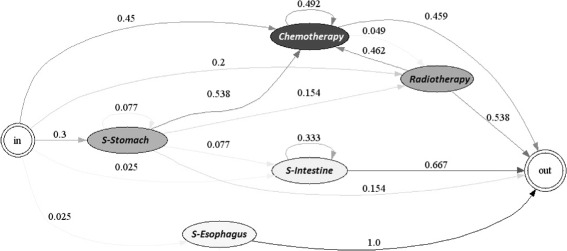
Chemotherapy/Radiotherapy Cycle for EPS 2, year 2010

**Fig. 13:**
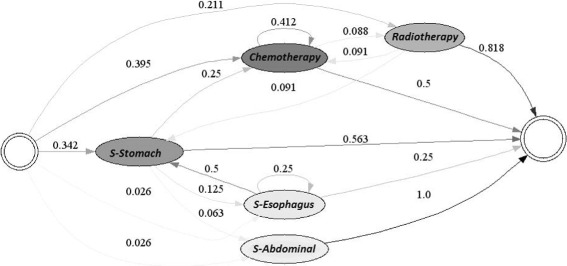
Chemotherapy/Radiotherapy Cycle for EPS 2, year 2011

Figures 12 and 13 present the chemotherapy and/or radiotherapy cycle for each of the years studied. It should be noted that cycles of chemotherapy and radiotherapy were observed in the treatment of the patients, since 49.2% of the patients that received chemotherapy at least once received chemotherapy again. The most common procedure for the patients was radiotherapy in 4.9% of the cases. Additionally, if a patient received radiotherapy, in 46.2% of the cases, the patient also received chemotherapy. It is noteworthy that none of the patients received radiotherapy in a consecutive, periodical manner.

In conclusion, the patients in EPS 2 have a higher probability of undergoing a second operation. This characteristic may be related to problems during the diagnostic step. Additionally, it does not apply radiotherapy consecutively and, in general, complies loosely with the available clinical guide. These elements can explain the position of EPS 2 in the efficient frontier.

## Discussion

This paper proposes an innovative approach applying Data Envelopment Analysis (DEA), and Process Mining to evaluate efficiency and quality in the treatment of gastric cancer. The aggregate strategy proposed was very useful in reducing the complexity in the models. Data Envelopment Analysis has been used by other authors to analysis hospital efficiency in Zambia [20] and Iran [31]. Our results show variation between different indicators and treatments used by insurance companies and are similar to other studies that are more focused on clinical guidelines analysis [32]. Other studies combined DEA with Malmquist indices to measure hospital productivity [33]. There is limitation in the database used in this study and other authors recommended a level 3 data base (the most comprehensive) with information from health program enrollment files, including when eligibility begins and ends [34]. The analysis should consider factors such as complexity and current regulations and guidelines in the treatment of different types of cancer. The use of specific information about the disease analyzed such as the stage of the cancer, studies focusing on IPS, or studies related to less complex diseases are suggested for future work. Efforts should be aimed at designing objective metrics that differentiate between efficient and inefficient entities in terms of procedures. Analyses oriented to identify hospitals or doctor’s behavior will be tackle using the same strategy. The analysis should consider factors such as complexity and current regulations and guidelines in the treatment of different types of cancer.

## Conclusions

This paper proposes a combined strategy, applying Data Envelopment Analysis (DEA), and Process Mining to evaluate efficiency and quality in the treatment of gastric cancer undertaken by health service providers in Colombia. Results showed that the method used is a useful approach to understand and tackle the problem. The analysis of the treatment of gastric cancer allowed us to find relevant information on the factors affecting a treatment process performed efficient or inefficiently. However, the complexity of the disease studied and the diversity of the health system, make it difficult to confirm the contribution obtained using the combined strategy proposed. The use of specific information about the disease analyzed such as the stage of the cancer, studies focusing on IPS, or studies related to less complex diseases are suggested for future work. On the other hand, it is important to note that the data preparation phase is very important to understand the data and to correct data quality problems in order to improve the quality of the results. In the same way, the aggregate strategy proposed was very useful in reducing the complexity in the models. Finally, the generalized analysis of the treatment for gastric cancer (the procedures were grouped by their role in the treatment rather than being studied as individual procedures), allowed us to improve the knowledge of the clinical treatment of a typical gastric cancer patient. As future work, efforts should be aimed at designing objective metrics that measure the differences between efficient and inefficient entities in terms of procedures. Additionally, the proposed strategy must be extended so that a preliminary analysis is carried to select the disease. The analysis should take into account factors such as complexity and current regulations and guidelines in the treatment of different types of cancer.

## Abbreviations

CHR: Committee on health regulation
CUPS: *Clasificación Única de Procedimientos en Salud*
DEA: Data envelopment analysis
DMU: Decision-making unit
EPS: *Entidades Promotoras de Salud.* ICD-10: International statistical classification of diseases and related health problems 10th revision
IPS: *Instituciones Prestadoras de Servicios de Salud*
MHW: Ministry of Health and Welfare
MI: Malmquist index
RIPS: *Registros Individuales de la Prestación de Servicios de Salud*
SOGCS: *Sistema Obligatorio de Garantía de Calidad en Salud*
